# Bioinformatics analyses of combined databases identify shared differentially expressed genes in cancer and autoimmune disease

**DOI:** 10.1186/s12967-023-03943-9

**Published:** 2023-02-10

**Authors:** Yuan Sui, Shuping Li, Xue-Qi Fu, Zhizhuang Joe Zhao, Shu Xing

**Affiliations:** 1 Edmond H. Fischer Signal Transduction Laboratory, School of Life Sciences, Jilin University, Changchun, 130012 China; 2 Department of Physiology, University of Oklahoma Health Sciences Center, Oklahoma City, OK 73104 USA; 3 Department of Pathology, University of Oklahoma Health Sciences Center, Oklahoma City, OK 73104 USA

**Keywords:** Tumorigenesis, Autoimmune genesis, Invasive ductal carcinoma, Systemic lupus erythematosus, STAT1, OAS1, OASL, PML, IFN-JAK-STAT signaling pathway

## Abstract

**Background:**

Inadequate immunity caused by poor immune surveillance leads to tumorigenesis, while excessive immunity due to breakdown of immune tolerance causes autoimmune genesis. Although the function of immunity during the onset of these two processes appears to be distinct, the underlying mechanism is shared. To date, gene expression data for large bodies of clinical samples are available, but the resemblances of tumorigenesis and autoimmune genesis in terms of immune responses remains to be summed up.

**Methods:**

Considering the high disease prevalence, we chose invasive ductal carcinoma (IDC) and systemic lupus erythematosus (SLE) to study the potential commonalities of immune responses. We obtained gene expression data of IDC/SLE patients and normal controls from five IDC databases (GSE29044, GSE21422, GSE22840, GSE15852, and GSE9309) and five SLE databases (GSE154851, GSE99967, GSE61635, GSE50635, and GSE17755). We intended to identify genes differentially expressed in both IDC and SLE by using three bioinformatics tools including GEO2R, the limma R package, and Weighted Gene Co-expression Network Analysis (WGCNA) to perform function enrichment, protein-protein network, and signaling pathway analyses.

**Results:**

The mRNA levels of signal transducer and activator of transcription 1 (STAT1), 2'-5'-oligoadenylate synthetase 1 (OAS1), 2'-5'-oligoadenylate synthetase like (OASL), and PML nuclear body scaffold (PML) were found to be differentially expressed in both IDC and SLE by using three different bioinformatics tools of GEO2R, the limma R package and WGCNA. From the combined databases in this study, the mRNA levels of STAT1 and OAS1 were increased in IDC while reduced in SLE. And the mRNA levels of OASL and PML were elevated in both IDC and SLE. Based on Kyoto Encyclopedia of Genes and Genomes pathway analysis and QIAGEN Ingenuity Pathway Analysis, both IDC and SLE were correlated with the changes of multiple components involved in the Interferon (IFN)-Janus kinase (JAK)-signal transducer and activator of transcription (STAT) signaling pathway.

**Conclusion:**

The expression levels of STAT1 and OAS1 manifest the opposite expression tendency across cancer and autoimmune disease. They are components in the IFN-JAK-STAT signaling pathway related to both tumorigenesis and autoimmune genesis. STAT1 and OAS1-associated IFN-JAK-STAT signaling could explain the commonalities during tumorigenesis and autoimmune genesis and render significant information for more precise treatment from the point of immune homeostasis.

**Supplementary Information:**

The online version contains supplementary material available at 10.1186/s12967-023-03943-9.

## Introduction

With functions of immune defense, surveillance, and homeostasis, immunity is a complicated biological system for maintaining the homeostasis in multicellular organisms. The immune system protects organisms from various pathogens and possibly harmful substances, eliminates invading pathogens, monitors interior milieu by recognizing and removing abnormal components, and maintains immunological homeostasis through immune tolerance and immune regulation. Any issues in the components comprising the immune system network may lead to multiple problems damaging human health, including hypersensitivity [1], pathogen infections [2], tumorigenesis [3], and autoimmune disorders [4].

Genetic changes can be well-explained by the tumorigenesis process [5], and the immune system is fully capable to clearing up the mutant cells/debris through the tumoricidal effects from natural killer (NK) cells and a series of boosting mechanisms to avoid tumorigenesis, such as the releasement of chemokines/pro-inflammatory cytokines in tumor microenvironment (TME), recruitment of conventional DCs (cDCs), differentiation of cytotoxic T cells (CTLs), help from cytotoxic neutrophils and anti-tumor T cells, alterations of pattern recognition, patrolling behavior of monocytes, and so on [6–10]. However, once cancer cells escape from the immune-surveillance, they tend to establish immune tolerance for them to survive in organisms, and immune responses are not able to eliminate these malignant cells, which lead to an uncontrollable growth.

Autoimmune disorders are characterized by abnormal activation in the immune system resulted from the loss of self-tolerance and disrupted immune homeostasis [11]. Under autoimmunity, the immune system attacks self-healthy tissues by autoreactive T cells and auto-antibodies causing persistently chronic inflammation and even multiple organ failures [12, 13]. The impacts from autoreactive T-lymphocytes to the initiation and/or progression of autoimmune disorder derive from CD4^+^ T cell activation, which is a coordinated procedure requiring multiple signals [14]. Furthermore, aberrant immune checkpoint proteins, programmed cell death protein 1/programmed cell death protein ligand 1 (PD-1/PD-L1), contribute to autoreactive immunity as well [15, 16]. Besides, humoral immunity plays a major role in biological damages during autoimmune disorders through the production of auto-antibody from plasma cells [17]. Although the antibody-secreting cells have a vital function in adaptive immunity, other B-lymphocytes regulate cellular immunity and affect immune response to a great extent as well during autoimmune disorders [18].

In terms of the onsets of disease, malignancies and autoimmune disorders are etiologically diverse, and the therapies for human diseases are more and more individualized and precisely targeted [19–21]. Although the function of immunity during the onset of these two processes appears to be distinct, the underlying mechanism is shared. To be specific, inadequate immune responses because of poor immune surveillance leads to tumorigenesis, while excessive immune responses due to breakdown of immune tolerance cause autoimmune genesis. To date, gene expression data for large bodies of clinical samples are available, but the commonalities of tumorigenesis and autoimmune genesis in terms of immune responses remains to be summed up. Hence, we were trying to construct a shared differentially gene expression profile to explore the commonalities between tumorigenesis and autoimmune genesis. The most common type of cancer is breast cancer accounting for 31% of new diagnosed cancers among the U.S. female population [22], and approximately 287,850 new cases of invasive breast cancer will be diagnosed among U.S. women in 2022 [23]. Invasive breast cancers are the most common histological subtypes of breast cancer [24], and invasive ductal carcinoma (IDC) are the most common type of breast cancer [25]. Systemic lupus erythematosus (SLE) is prevalent in 20–150 per 100,000 people, and higher prevalence and greater relevance of organ damaging are common among people of multiple ancestries in United State [26]. In terms of the distribution of the SLE population, the majority of patients with SLE are women of childbearing age [27], overlapping with the onset of IDC. Hence, we conducted an IDC-SLE shared differentially gene expression analysis to explore the potential commonalities and connections between tumorigenesis and autoimmune genesis to explain the differences of their immune responses.

## Methods

### IDC data collection and differentially expressed genes (DEGs) and hub genes identification

**Fig. 1:**
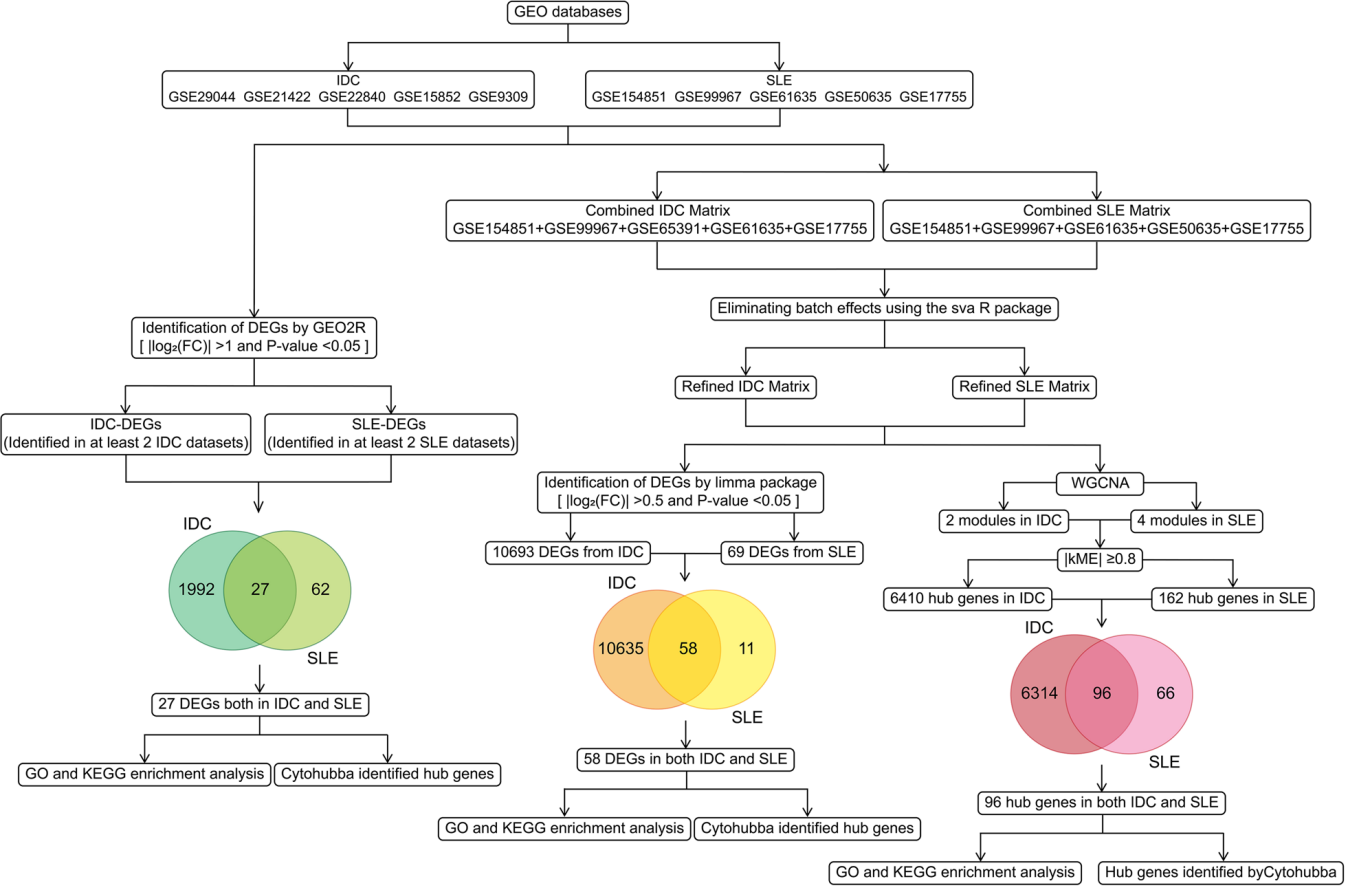
Flowchart of data collection and analyses

**Fig. 2:**
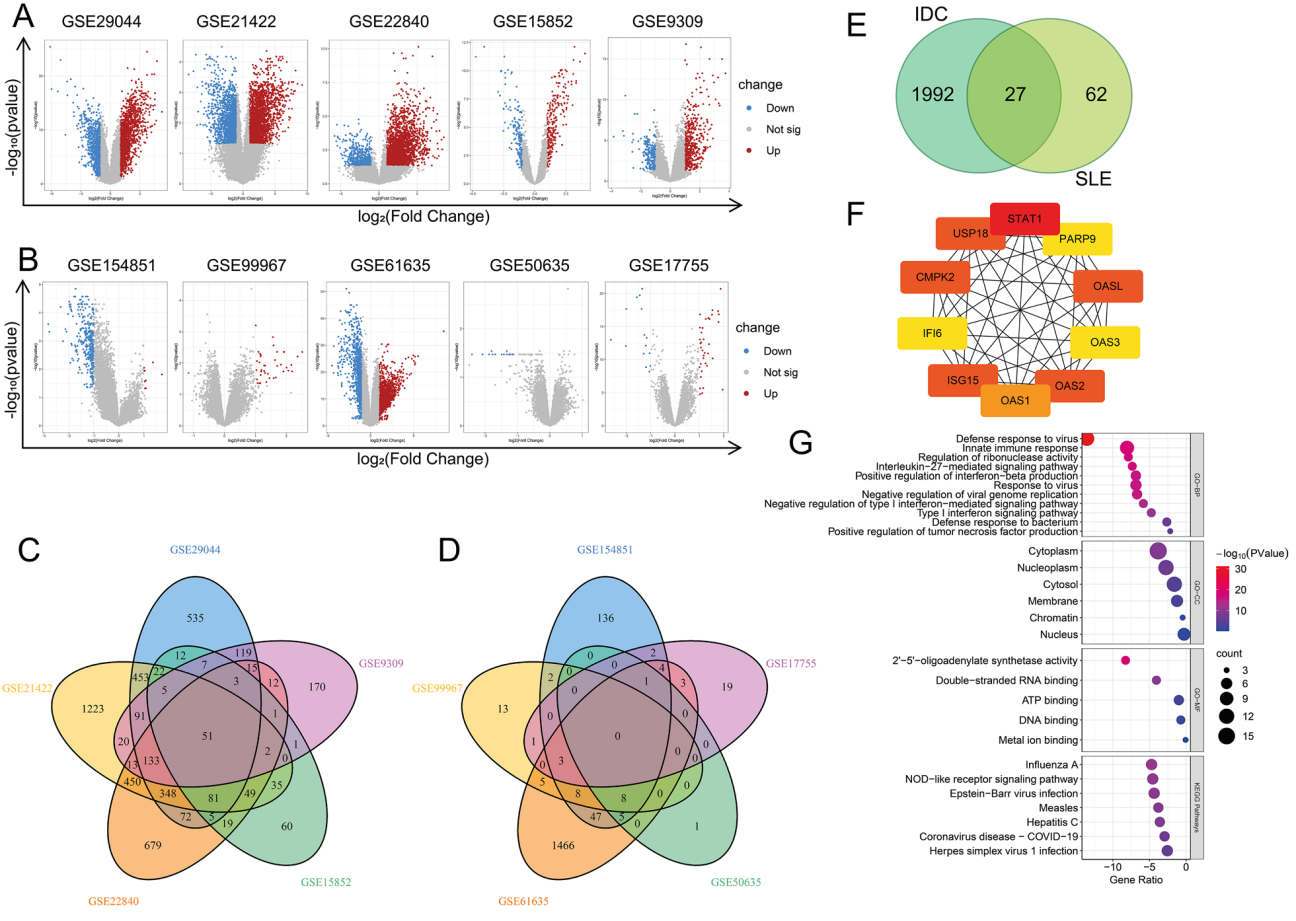
Identification and analyses of DEGs in IDC and SLE by GEO2R. **A** and **B** The volcano plots illustrate the differential gene expressions in five IDC (A) and five SLE (B) datasets. The negative log_10_-transformed p values (Y axis) are plotted against the average log_2_ fold changes (X axis) in gene expressions. Identified DEGs are shown in red (log_2_ (FC) > 1) and blue (log_2_ (FC) < -1). The p value cut-off is < 0.05. **C** and **D** Venn diagrams show intersected DEGs among five IDC (C) and five SLE (D) datasets. The area is proportional to the number of genes. **E** Venn diagram showing the total and intersected numbers of DEGs in IDC and SLE. **F** PPI network of top 10 genes out of the 27 overlapping DEGs based on BC values obtained from Cytoscape plug-in Cytohubba. Colors represent BC values from high (red) to low (yellow). **G** GO and KEGG enrichment analysis of 27 DEGs shown in **E**

**Fig. 3:**
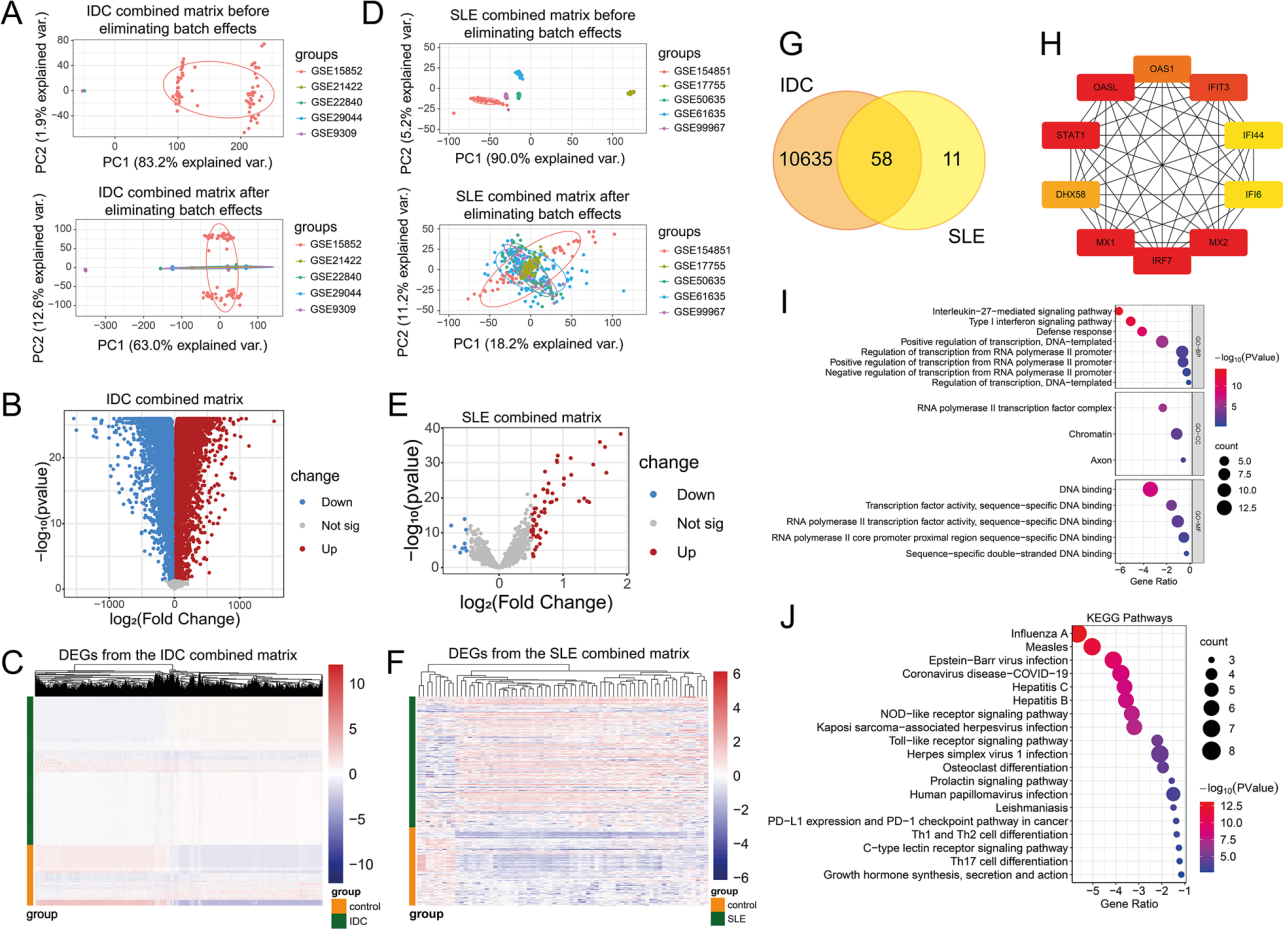
Identification and analyses of DEGs from the combined IDC and SLE datasets by differential gene analysis of the limma R package. **A** and **D** Principal component analysis of five IDC(A)/SLE(D) datasets before (upper panel) and after (low panel) elimination of batch effects. **B** and **E** The volcano plots of the genes from the combined matrix from five IDC(B)/SLE(E) studies after eliminating batch effects, and DEGs (p-value < 0.05) were plotted in red (log_2_ (FC) > 0.5) and blue (log_2_ (FC) < -0.5). **C** and **F** The heatmap of the DEGs in IDC(C)/SLE(F) expression in normal controls and IDC/SLE patients. The DEGs in IDC/SLE were the DEGs shown in (B)/(E) with red or blue. **G**. Venn diagram showing the total and intersected numbers of DEGs in IDC and SLE. **H** PPI network of top 10 genes out of the 58 overlapping DEGs based on BC values obtained from Cytoscape plug-in Cytohubba. Colors represent BC values from high (red) to low (yellow). **I** GO enrichment analysis based on the 58 DEGs shown in (G). **J** KEGG enrichment analysis based on the 58 DEGs shown in (**G**)

**Fig. 4:**
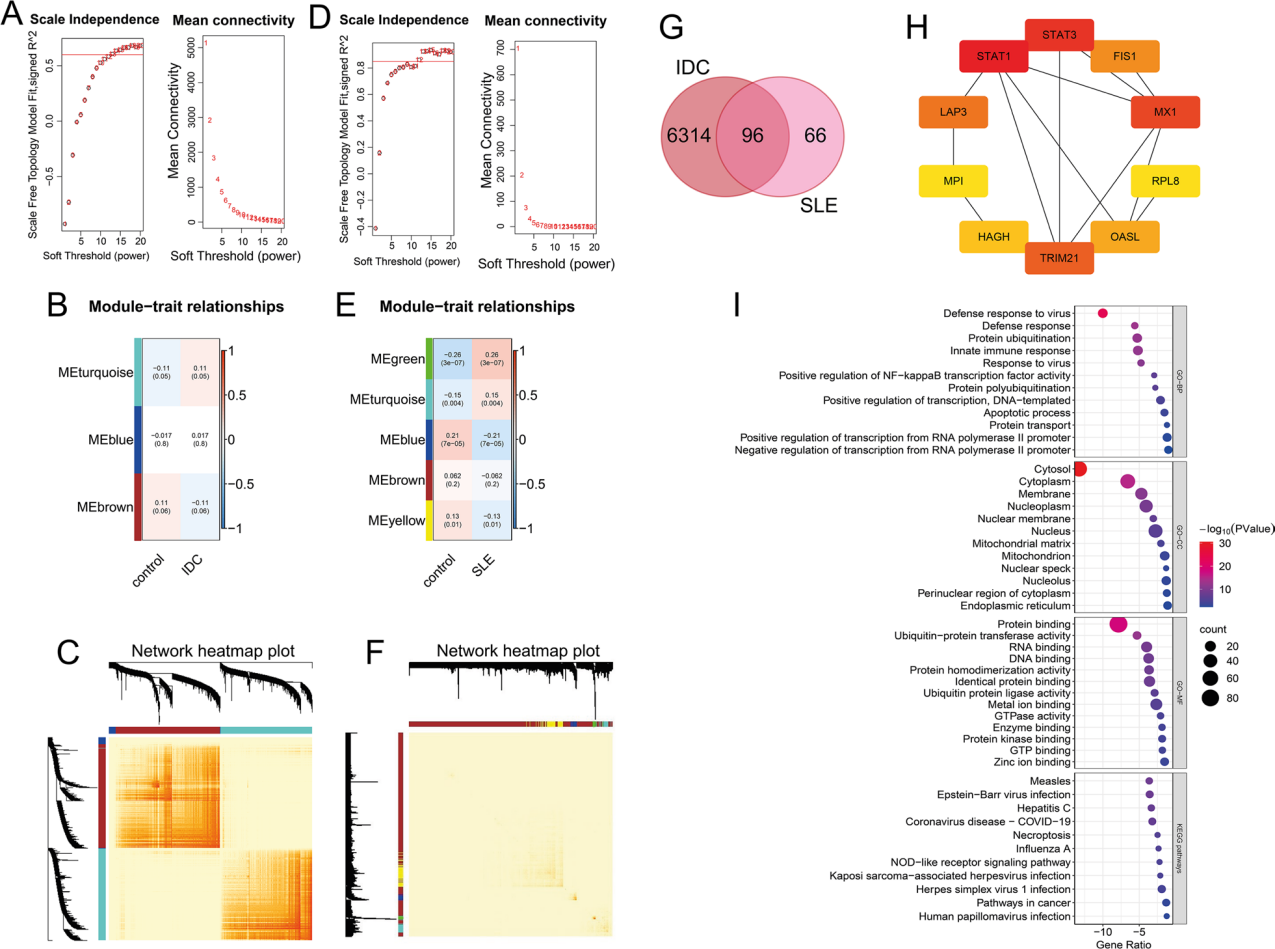
Weighted co-expression network analysis for identification and analyses of hub genes from the combined IDC and SLE datasets. **A** and **D** The left panel was the analysis of the scale-free fit index with multiple soft-thresholding powers (β), and the right panel was the analysis of the mean connectivity with multiple soft-thresholding powers. Both of them were based on the combined gene expression matrix of IDC(A)/SLE(D) from the five IDC/SLE studies. **B** and **E** Correlations between MEs and groups indicating the module-trait associations. Every row represented a ME, and every column represented the group. The groups of (B) contain control and IDC patients. The groups of (E) contain control and SLE patients. **C** and **F** The heatmaps with topology showing gene network of IDC(C)/SLE(F). The rows and columns represented gene list. The gene dendrogram and module assignment were shown at the left and top. **G** Venn diagram showing the total and intersected numbers of hub gene (|kME|≥ 0.8) in IDC and SLE. **H** PPI network of top 10 genes out of the 96 overlapping hub genes based on BC values obtained from Cytoscape plug-in Cytohubba. Colors represent BC values from high (red) to low (yellow). **I** GO and KEGG enrichment analysis based on the 96 hub genes shown in (**G**)

To study tumorigenesis process, we attempted to include the IDC studies that contain most IDC patients paired with adjacent normal tissue or healthy breast as control. We downloaded gene expression matrixes and clinical information (age, gender, and disease severity) of five selected IDC studies including GSE29044, GSE21422**,** GSE22840, GSE15852, and GSE9309 from Gene Expression Omnibus (GEO, https://www.ncbi.nlm.nih.gov/gds). These contained 97 controls and 237 IDC samples (Table1). We used Grade to evaluate the disease severity of IDC: breast cancers were categorized as luminal A (ER-positive and/or PR-positive and HER2- and either histologic grade 1 or 2); luminal B (ER-positive and/or PR-positive and HER2+ or ER-positive and/or PR-positive, HER2- and grade 3); HER2 (ER-negative and PR-negative and HER2+); and triple negative (ER-, PR-, and HER2-) [28]. As the flowchart (Fig. 1) shows, we were trying to identify the crucial genes during IDC initiation with three bioinformatics tools (GEO2R, the limma R package, and WGCNA). The first method uses GEO2R (https://www.ncbi.nlm.nih.gov/geo/geo2r/) to figure out DEGs. The genes satisfying |log_2_(FC)|> 1 and the p-value < 0.05 were statistically significant, and all of the DEGs from five IDC studies, which were identified in at least two IDC studies, were considered as DEGs in IDC within this method (Fig. 2). The Venn diagram including DEGs from five IDC studies was used to show mathematical or logical connections between different collections of elements. The second method combined five gene expression matrixes from the five IDC studies to obtain a combined matrix for differential gene analysis. Then, we performed differential gene analysis in the combined matrix with the limma R package [29] after eliminating batch effects by the sva package [30]. When |log_2_(FC)|> 0.5 and p-value < 0.05 were satisfied, the gene was considered as being statistically significant in the combined matrix (Fig. 3). The third method was the identification of modules by weighted gene co-expression network analysis (WGCNA) in the IDC combined matrix. WGCNA was performed on 10,949 genes by using the WGCNA R package [32]. A soft threshold of β = 12 (R^2^ > 0.6) (Fig. 4A) and a minimum module size of 30 was selected to yield three modules (Fig. 4B). The Pearson’s correlation coefficients were calculated between the samples and within each module (Fig. 4B). The genes with absolute module eigengene-based connectivity (kME) values of no less than 0.8 were regarded as hub genes.

**Table 1:** IDC and SLE expression profile data sets from GEO database

Database (IDC/SLE)	Dataset ID	Platform	Number		Type	Tissue (IDC/Normal)	Age (years)		Gender	Disease severity
			IDC/SLE	Normal						
IDC	GSE29044	GPL570	67	36	mRNA	Tumor/Normal breast	20–35	10	NA	▲Grade N/A: 7 Grade1: 3 Grade2: 52 Grade3: 41
							36–45	46		
							46–55	16		
							> 55	30		
							62	1		
IDC	GSE21422	GPL570	5	5	mRNA	Tumor/Healthy breast	NA		NA	NA
IDC	GSE22840	GPL570	14	4	mRNA	Tumor/Normal breast	NA		NA	NA
IDC	GSE15852	GPL96	35	43	mRNA	Tumor/Normal breast	52(47,54)		NA	Normal: 43 Grade1: 5 Grade2: 18 Grade3: 12
IDC	GSE9309	GPL887	116	9	mRNA	Tumor/Breast nontumor part	NA		Female: 125	NA
									Male: 0	
SLE	GSE154851	GPL16699	38	32	mRNA	Whole blood	33(30,36)		Female: 68	NA
									Male: 2	
SLE	GSE99967	GPL21970	42	17	mRNA	Peripheral blood	31(28.9,34)		Female: 48	SLIDAI of 42 patients: 12(10,16)
									Male: 11	
SLE	GSE61635	GPL570	99	30	mRNA	Blood	NA		NA	NA
SLE	GSE50635	GPL6244	33	16	mRNA	Whole blood	46(43,53)		Female: 49	NA
									Male: 0	
SLE	GSE17755	GPL1291	22	45	mRNA	Peripheral blood	34(31,39)		Female: 44	NA
									Male:23	

Age were presented by [median (Lower 95% CI, Upper 95% CI)] or sample number. SLIDAI of 42 patients were presented by [median (Lower 95% CI, Upper 95% CI)]. ▲Cancers were categorized as luminal A (ER-positive and/or PR-positive and HER2- and either histologic grade 1 or 2); luminal B (ER-positive and/or PR-positive and HER2 + or ER-positive and/or PR-positive, HER2- and grade 3); HER2 (ER-negative and PR-negative and HER2 +); and triple negative (ER-, PR-, and HER2-) according to original paper (Colak D, et al. PloS one. 2013;8(5):e63204). IDC, Infiltrating Ductal Carcinoma; SLE, Systemic Lupus Erythematosus; GEO, Gene Expression Omnibus; SLIDAI, Systemic Lupus Erythematosus Disease Activity Index; NA, not available

### SLE data collection and DEGs and hub genes identification

To study autoimmune genesis process, we attempted to include the SLE studies that contain SLE patients and a considerable number of normal samples as control. We downloaded gene expression matrixes and clinical information (age, gender, and disease severity) of five selected SLE studies including GSE154851, GSE99967, GSE61635, GSE50635, and GSE17755 from Gene Expression Omnibus (GEO, https://www.ncbi.nlm.nih.gov/gds). These contained 140 controls and 234 SLE samples (Table 1). We used systemic lupus erythematosus disease activity index (SLIDAI) to evaluate the disease severity of SLE. As the flowchart (Fig. 1) shows, we intended to identify crucial genes during SLE initiation with three bioinformatics tools (GEO2R, the limma R package, and WGCNA). The first method was using GEO2R (https://www.ncbi.nlm.nih.gov/geo/geo2r/) to figure out DEGs. The genes satisfying |log_2_(FC)|> 1 and the p-value < 0.05 were statistically significant, and all of the DEGs from five SLE studies, which were identified in at least two SLE studies, were considered as DEGs in SLE within this method (Fig. 2). The Venn diagram including the DEGs from five SLE studies was used to show mathematical or logical connections between different collections of elements. The second method combined five gene expression matrixes from five SLE studies to obtain a combined matrix for differential gene analysis. We performed differential gene analyses in the combined matrix by the limma R package [29] after eliminating batch effects by the sva package [30]. When |log_2_(FC)|> 0.5 and p-value < 0.05, the gene was considered as being statistically significant in the combined matrix (Fig. 3). The third method was to identify modules by WGCNA in the SLE combined matrix. WGCNA was performed on 3,857 genes by using the WGCNA R package [32]. A soft threshold of β = 9 (R^2^ > 0.85) (Fig. 4D) and a minimum module size of 30 was selected to yield five modules (Fig. 4E). The Pearson’s correlation coefficients were calculated between the samples and within each module (Fig. 4E). The genes with absolute eigengene-based connectivity (kME) values of no less than 0.8 were regarded as hub genes.

### Principal component analysis (PCA)

Before and after batch effects elimination, PCA were conducted by using the ggbiplot R package.

### Function enrichment analysis

The DEGs and/or the hub genes screened from the three bioinformatics methods were subjected to Gene Ontology (GO) enrichment analysis and Kyoto Encyclopedia of Genes and Genomes (KEGG) pathway analysis by using the Annotation, Visualization and Integrated Discovery (DAVID) database (https://david.ncifcrf.gov/) [34]. A p-value < 0.05 under the hypergeometric test was considered significant. The final visualizations of function enrichment analysis were performed by using the ggplot2 R package.

### Protein-protein interaction (PPI) network analysis

The gene information in PPI networks was downloaded from the STRING database (https://string-db.org/). The hub genes were screened with Cytoscape plug-in Cytohubba according to the betweenness centrality (BC) of the gene in the PPI network of the three methods. The calculation of BC was conducted by Cytoscape plug-in Cytohubba and Cytoscape plug-in CytoNCA [36]. All PPI networks were operated within Cytoscape (https://cytoscape.org/) [36].

### Gene identification by using three bioinformatics tools

**Fig. 5:**
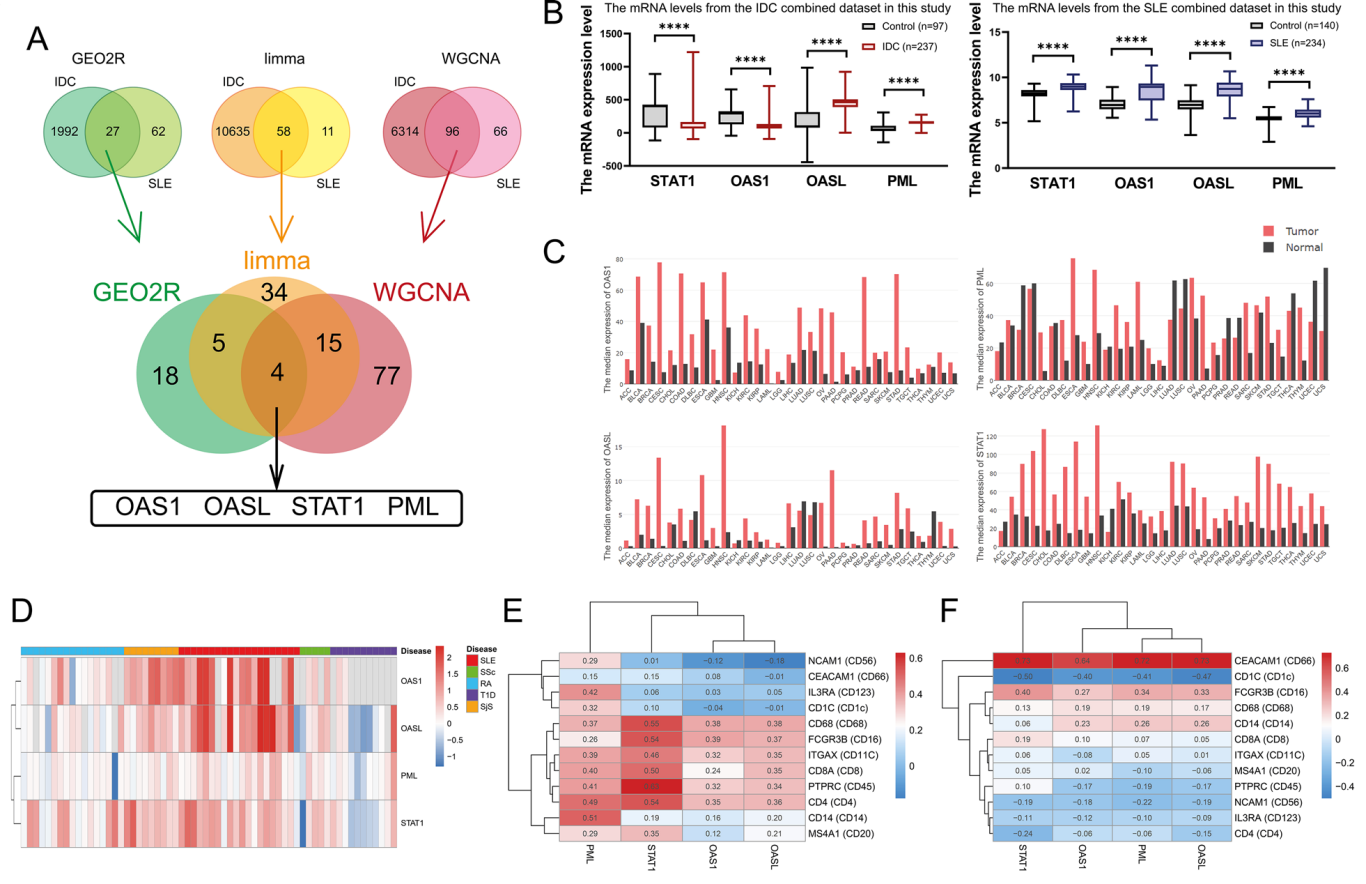
Gene identification by using three bioinformatics methods and their expression profiles in multiple cancers and autoimmune diseases and their correlation with immune cell markers in BRCA and SLE. **A** Schematic plot of the combination with three bioinformatics tools. **B** The mRNA levels of STAT1, OAS1, OASL, and PML from the combined IDC (left panel) and SLE (right panel) datasets in this study. **C** Bar plots from GEPIA database with the gene expression profile (OAS1, OASL, PML, and STAT1) across multiple types of tumor samples and paired normal tissues. The height of bar represented the median expression of certain tumor type or normal tissue, and the horizontal axis indicated tumor names. **D** The heatmap from ADEx database indicating the values of fold change of OAS1, OASL, PML, and STAT1 between multiple types of autoimmune diseases and paired normal individuals. **E** and **F** The heatmaps represent the Spearman’s correlation coefficients (R^2^) between DEGs expressions (OAS1, OASL, PML, and STAT1) and multiple immune cell marker genes (the encoded protein by the gene) in BRCA tumor (**E**) or SLE (**F**). The Spearman’s correlation coefficients (R^2^) were labeled at nodes of every two genes in heatmaps

**Fig. 6:**
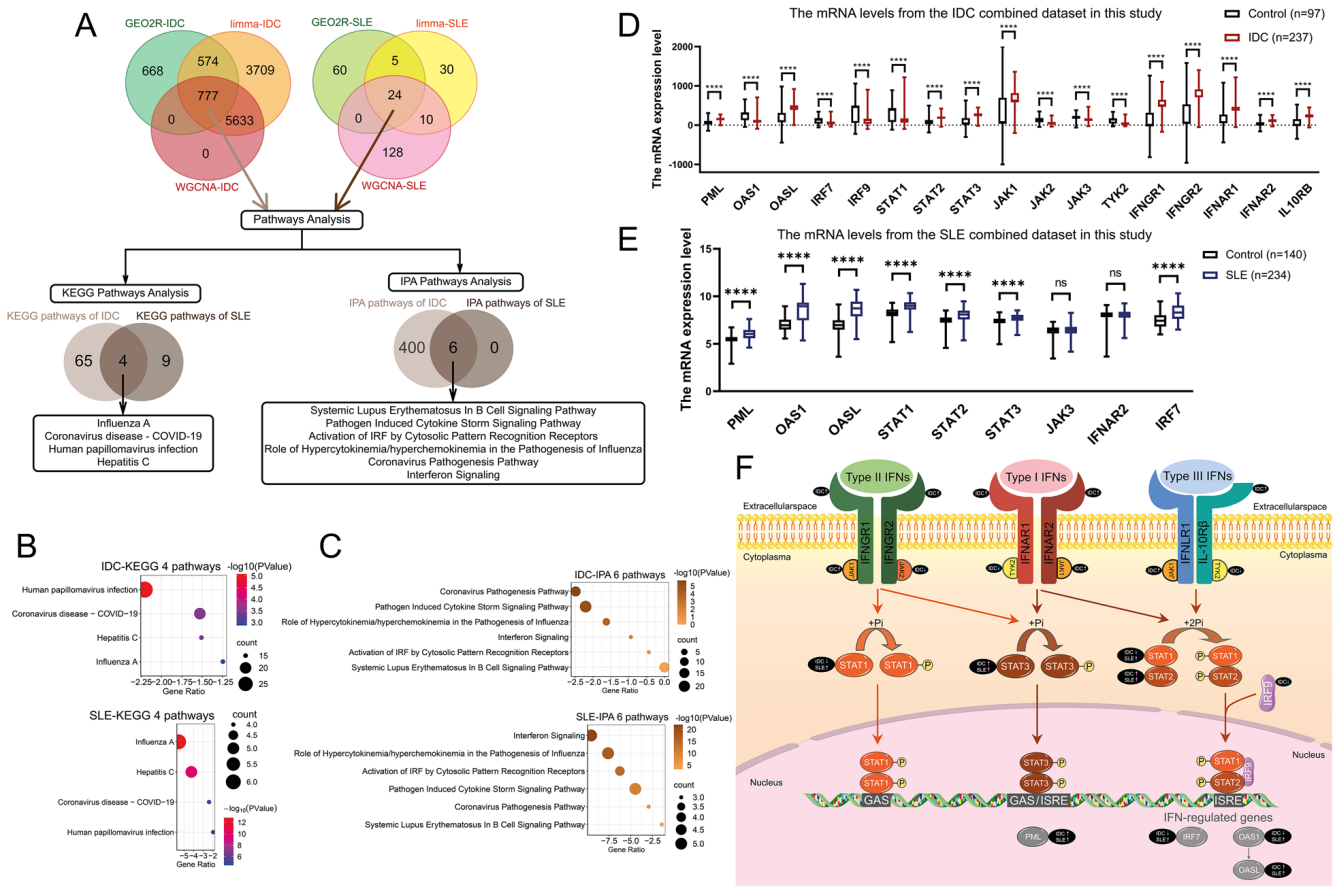
Signaling pathway analyses in IDC and SLE. **A** Schematic plot of signaling pathways analyses. **B** The four overlapped KEGG pathways from 24 SLE specific genes and 777 IDC specific genes. **C** The six overlapped IPA pathways from 24 SLE specific genes and 777 IDC specific genes. **D** and **E** The mRNA levels of the components in IFN-JAK-STAT pathway from the combined IDC (D) and SLE (E) datasets in this study. **F** Schematic diagram of IFN-JAK-STAT pathway changes in IDC and SLE

We identified four shared differentially expressed genes in IDC and SLE by using the three different methods (Fig. 5A). The mRNA levels of signal transducer and activator of transcription 1 (STAT1), 2'-5'-oligoadenylate synthetase 1 (OAS1), 2'-5'-oligoadenylate synthetase like (OASL), and PML nuclear body scaffold (PML), and the multiple components in Interferon (IFN)-Janus kinase(Jak) -signal transducer and activator of transcription (STAT) (IFN-JAK-STAT) signaling pathway from the combined matrix of IDC and SLE were presented as box plots (Fig. 5B, Fig. 6D, and Fig. 6E). Min to Max were used for statistical description. Data were analyzed for significance with two-tailed nonparametric test. The p-values < 0.05 were considered statistically significant. The visualization of box plots and statistical analyses were conducted in GraphPad Prism 9.0 (GraphPad Software Inc.,LLC).

### Expression profiles of STAT1, OAS1, OASL, and PML in multiple cancers and autoimmune diseases

We downloaded and analyzed the bar plots with the gene expression of STAT1, OAS1, OASL, and PML in multiple malignancies and paired normal tissues (Fig. 5C) from the GEPIA2 database (http://gepia2.cancer-pku.cn/#index) [37]. We downloaded and analyzed the heatmap with the fold change of STAT1, OAS1, OASL, and PML between multiple autoimmune diseases and paired normal individuals (Fig. 5D) from the ADEx database (https://adex.genyo.es/) [38].

### Spearman’s correlation analysis of STAT1, OAS1, OASL, and PML with immune cell markers in BRCA and SLE

Here we defined that CD45 marked leukocytes, CD66 marked neutrophils, CD14 marked monocytes, CD68 marked macrophages, CD16 marked monocytes/macrophages/dendritic cells (DCs), CD11C marked monocytes/DCs, CD123 marked plasmacytoid dendritic cells (pDCs), CD1C marked DC, CD56 marked NK cells, CD4 marked CD4^+^T cells, CD8 marked CD8^+^T cells, and CD20 marked B cells. The Spearman’s correlation coefficients (R^2^) of the tumor tissue expression between STAT1, OAS1, OASL, and PML and multiple immune cell markers in breast invasive carcinoma (BRCA) were obtained from GEPIA2 database (http://gepia2.cancer-pku.cn/#index) [37]. The gene expression matrix including the mRNA level of STAT1, OAS1, OASL, PML, and multiple immune cell markers from 292 SLE patients’ whole blood samples were downloaded from GSE45291 (https://www.ncbi.nlm.nih.gov/geo/query/acc.cgi?acc=GSE45291) for calculating Spearman’s correlation coefficients (R^2^). The Spearman’s correlation analysis were performed in GraphPad Prism 9.0. The Spearman’s correlation coefficients (R^2^) were presented by heatmaps (Fig. 5E and Fig. 5F) conducted by the pheatmap R package.

### QIAGEN Ingenuity Pathway Analysis (QIAGEN IPA)

The fold changes and p-values of the DEGs were identified from the IDC/SLE combined expression matrix by the limma R package [29]. The DEGs were uploaded to the QIAGEN IPA server and performed with core expression analysis as previously described [40].

### Software application

R is a free software environment for statistical computing and graphics and can be downloaded from https://cran.r-project.org/mirrors.html. RStudio is an integrated development environment for R, a programming language for statistical computing and graphics, and the RStudio Desktop Open Source Edition (AGPL v3) can be downloaded from https://posit.co/download/rstudio-desktop/. R version 4.1.3 and RStudio version 2022.02.3 were used in this study. Venn diagram in this study were drawn by the VennDiagram R package [41] or the Venn webtools website (http://bioinformatics.psb.ugent.be/webtools/Venn/). GraphPad Prism 9.0 (GraphPad Software Inc.,LLC) was purchased by The University of Oklahoma. Ingenuity IPA license was purchased from QIAGEN (https://digitalinsights.qiagen.com/products-overview/discovery-insights-portfolio/analysis-and-visualization/qiagen-ipa/).

## Results

### The DEGs/hub genes identified by three bioinformatics methods enriched in the biological processes and pathways relating to virus defense, virus infection, and interferon

By using GEO2R, we defined |log_2_(FC)|> 1 and the p-value < 0.05 as the threshold in five databases of IDC and SLE according to the numbers of overlapped DEGs (Additional file 2: Table S6) in the method. We identified 2019 DEGs in IDC (Fig. 2C), 89 DEGs in SLE (Fig. 2D), and 27 DEGs both in IDC and SLE (Fig. 2E). The top 10 screened BC genes from the 27 DEGs were STAT1, IRF7, OAS1, OAS2, USP18, CMPK2, OASL, ISG15, IFI6, and OAS3 (Fig. 2F), and their ranks and scores were shown in Additional file 1 Table S1. The DEGs are enriched in the biological processes relating to virus defense and interferon such as Defense response to virus, Innate immune response, and Response to virus, and pathways relating to virus infection such as Influenza A and Epstein-Barr virus infection (Fig. 2G).

We also combined five IDC/SLE database for differential gene expression analysis. The batch effects were eliminated in the combined IDC matrix (Fig. 3A) and the combined SLE matrix (Fig. 3D). We defined |log_2_(FC)|> 0.5 and the p-value < 0.05 as the threshold in five databases of IDC and SLE according to the numbers of DEGs (Additional file 3: Table S7). The results from differential gene analyses indicated that the DEGs in IDC (Fig. 3B) and SLE (Fig. 3E) differentially expressed between the control and patients (Fig. 3C and Fig. 3F). There were 58 DEGs were identified from both IDC and SLE (Fig. 3G). The top 10 screened BC genes from the 58 DEGs were IRF7, MX1, STAT1, OASL, MX2, IFIT3, OAS1, DHX58, IFI6, and IFI44 (Fig. 3H), and their ranks and scores were shown in Additional file 1 Table S2. The DEGs enriched in the biological processes relating to defense and interferon such as Type I interferon signaling pathway and Defense response (Fig. 3I), and the pathways relating to virus infection and inflammation reactions such as Influenza A, Measles, Th17 cell differentiation (Fig. 3J).

The combined IDC/SLE database were used for WGCNA. In combined IDC expression matrix, there were 10,949 genes for WGCNA. As the sample tree in Additional file 6: Figure S3 shows, there were no outliers (the samples with obvious abnormal gene expression) in IDC. So, no samples were removed (97 controls and 237 IDC samples). In combined SLE expression matrix, we removed GSM2666765 (control) and GSM4681556 (SLE), because they were outliers in Additional file 5: Figure S4. So, there were 3,857 genes and 372 samples (139 controls and 233 SLE samples) for subsequent WGCNA. From the WGCNA results of IDC, MEturquoise and MEbrown were significantly different (p < 0.1), and they were considered as the interesting modules in IDC (Fig. 4B). From the WGCNA results of SLE, MEgreen, MEturquoise, MEblue, and MEyellow were significantly different (p < 0.1), and they were considered as the interesting modules in SLE (Fig. 4E). There were 6,410 genes in IDC and 162 genes in SLE that identified as hub genes from these interesting modules. There were 96 hub genes were identified from both IDC and SLE (Fig. 4G). The top 10 screened BC genes from the 96 hub genes were STAT1, STAT3, MX1, TRIM21, LAP3, FIS1, OASL, HAGH, MPI, and RPL8 (Fig. 4H), and their ranks and scores were shown in Additional file 1: Table S3. The DEGs enriched in the biological processes relating to virus such as Defense response to virus and Response to virus, and the pathways relating to virus infection such as Measles and Epstein-Barr virus infection (Fig. 4I).

### STAT1, OAS1, OASL, and PML were identified as the shared differentially expressed genes in IDC and SLE. And STAT1 and OAS1 indicated the opposite expressed tendency across IDC and SLE

As the schematic plot shows in Fig. 5A, STAT1, OAS1, OASL, and PML differentially expressed on both IDC and SLE according to the three bioinformatics methods (GEO2R, the limma R package, and WGCNA). From the combined databases in this study, STAT1 and OAS1 were increased in IDC while reduced in SLE manifesting opposite expression tendency on IDC and SLE (Fig. 5B). And OASL and PML were elevated in both IDC and SLE (Fig. 5B). The shared differentially expressed genes in IDC and SLE enriched in the biological processes and pathways relating to virus defense, virus infection, and interferon (Additional file 4: Figure S1).

### STAT1, OAS1, OASL, and PML increased in most of malignancies. In autoimmune diseases, OAS1, OASL, and STAT1 increased while PML was not significant. Their mRNA levels correlated with immune cell markers of innate immunity in BRCA and SLE

Regarding the verification in malignancy, we found that the gene expression of STAT1, OAS1, OASL, and PML were increased in most of the malignancies in comparison with paired normal tissues (Fig. 5C). Regarding the verification of autoimmune diseases, the heatmap indicated that the fold changes of OAS1, OASL, and STAT1 between multiple autoimmune diseases and paired normal individuals were more than one in SLE, systemic sclerosis (SSc), and Sjögren's syndrome (SjS), especially in SLE, while the fold changes of PML were near zero, which means no significant difference between the patients with autoimmune disease and normal individuals (Fig. 5D). According to correlation analysis, expression of STAT1, OAS1, OASL, and PML on BRCA tumor tissue were positively correlated with immune cell markers from both innate immunity and adaptive immunity (Fig. 5E), while expression of STAT1, OAS1, OASL, and PML in whole blood of SLE was positively correlated with most of immune markers in innate immunity (Fig. 5F).

### The DEGs/hub genes in IDC and SLE are enriched in the IFN-JAK-STAT pathway

As the schematic plot in Fig. 6A shows, we identified 777 genes specific in IDC and 24 genes specific in SLE using the three bioinformatics methods (GEO2R, the limma R package, and WGCNA). The 69 KEGG pathways from the 777 genes in IDC (Additional file 5: Figure S2A) and 13 KEGG pathways from the 24 genes in SLE (Additional file 5: Figure S2A) were identified, while the 406 IPA pathways from the 777 genes in IDC and six IPA pathways from the 24 genes in SLE (Fig. 6A) were identified after filtering the pathway with the gene counts less than three (Additional file 1: Table S4 and Table S5). There were four overlapped KEGG pathways from IDC and SLE (Fig. 6B), and there were six overlapped IPA pathways from IDC and SLE (Fig. 6C). These intersected pathways were IFN-JAK-STAT pathway-based pathways or biological processes, such as viral infections and immune signaling (Fig. 6B and Fig. 6C).

According to the combined IDC expression matrix, the mRNA level of PML, OASL, STAT2, STAT3, JAK1, IFNGR1, IFNGR2, IFNAR1, IFNAR2, and IL10RB increased, while the mRNA level of OAS1, IRF7, IRF9, STAT1, JAK2 JAK3, and TYK2 decreased in IDC (Fig. 6D). According to the combined SLE expression matrix, the mRNA level of PML, OAS1, OASL, STAT1, STAT2, STAT3, and IRF7 increased in SLE (Fig. 6E). Hence, the mRNA levels of components in IFN-JAK-STAT pathway changed in IDC and SLE (Fig. 6F), which indicated that the IFN-JAK-STAT pathway was both relevant to the initiation of IDC and SLE.

## Discussion

In the present study, we found the mRNA levels of STAT1, OAS1, OASL, and PML were differential expressed in both IDC and SLE by using three different bioinformatics tools of GEO2R, the limma R package and WGCNA. From the combined databases in this study, the mRNA levels of STAT1 and OAS1 were increased in IDC while reduced in SLE manifesting the opposite expression tendency across cancer and autoimmune disease. And the mRNA levels of OASL and PML were elevated in both IDC and SLE. According to pathway analysis of KEGG and IPA, both IDC and SLE were correlated with the changes of multiple components involved in the IFN-JAK-STAT pathway.

The main results of this study are consistent with earlier findings. In IDC, we found that the mRNA levels of OAS1 and STAT1 were downregulated (Fig. 5B, Fig. 6D and Fig. 6F), which has also been reported in other cancers [42–44]. Moreover, OAS1 negatively regulated the expression levels of interferon responsive genes (IRGs), including OASL [45, 46], which agrees with our data that the mRNA levels of STAT1 and OAS1 were both downregulated in IDC. Additionally, our results indicated the upregulation of PML in IDC with downregulated/deficient STAT1. It was shown that interferon responsive pathways redirected toward STAT3 responses in the absence of STAT1, and STAT3 was able to regulate PML expression [47, 48]. In SLE, the mRNA level of STAT1, OAS1, OASL, and PML were upregulated (Fig. 5B, Fig. 6E and Fig. 6F), which were consistent with previous publications [49, 50]. Viral infections generated IFNs, which were accompanied by the transcription of OASL, and activated IFN responsive pathways after upregulating IFN regulated genes including OAS1 and STAT1. And enhanced IFN responsive pathways in viral infections tend to jeopardize the immune balance thereby developing autoimmune disorders such as SLE [51]. Also, other studies showed that STAT1 was essential in innate immunity and positively correlated with the susceptibility of viral infections [50]. Furthermore, both mRNA and protein levels of STAT1 presented positive causal relationship with SLE [49, 52, 53]. Hence, in patients with IDC, STAT1 and OAS1 downregulation may change transcriptional regulation in IFN responses thereby facilitating tumorigenesis. In patients with SLE, increased interferon signaling, especially STAT1 and OAS1, may facilitate disease initiation.

The IFN-JAK-STAT pathway has been well-studied in the fields of oncology and autoimmunity. And we identified it from the differences between the normal and patients based on clinical samples in IDC and SLE. The IFN-JAK-STAT pathway plays pivotal roles in anti-viral immune defense [54]. Since IFN was firstly described as a crucial molecule in blocking viral infections [55], the IFN family was successively considered as a central components contributing host-innate defense against viral pathogens [54]. The JAK/STAT signaling pathway transmit extracellular chemical signals to the nucleus for downstream gene transcription. The JAK/STAT pathway is comprised of cell receptors, JAK proteins (JAK1-3 and TYK2), and STAT proteins (1–4, 5a, 5b, and 6) [56]. The tyrosine residues on the tail of receptors are phosphorylated after binding of IFNs [57]. The phosphorylation of receptors activates JAKs to phosphorylate STATs by rendering an accessible binding site for STAT proteins due to the creation of a phosphotyrosine-based motif [54]. The STAT1-STAT1 homodimer or the STAT1-STAT2 heterodimer binding with a cytoplasmic IRF9 form the complex and translocate from cytosol into the nucleus to bind to IFN-stimulated response element (ISRE) to trigger the transcription of the IFN-stimulated genes (ISGs) and exerting IFN-α effects [58]. From our results, on one hand, the mRNA levels of STAT1, OAS1, OASL, and PML were both differentially expressed in IDC and SLE (Fig. 5A) by three bioinformatics tools including GEO2R (Fig. 2), the limma R package (Fig. 3), and WGCNA (Fig. 4). Furthermore, functional enrichment analysis in each mode indicated the association of diseases (IDC and SLE) with virus infections and viral defense responses (Fig. 2G, Fig. 3I, Fig. 3J, and Fig. 4I). Furthermore, the mRNA levels of STAT1, OAS1, OASL, and PML correlated with the markers of innate immunity in IDC and SLE (Fig. 5E and Fig. 5F). On the other hand, pathway analysis indicated that the onsets of IDC and SLE both were correlated with the changes of multiple components in the IFN-JAK-STAT signaling pathway, including STAT1, OAS1, OASL, and PML (Additional file 5: Figure S2, Additional file 1: Table S4, and Table S5). A schematic plot indicated the related components in interferon response pathways (Fig. 6F) and their changes in IDC/SLE. To summarize, three types of IFNs (IFN-type I, type II, and type III) binding with corresponding receptors phosphorylate STAT1 via JAK1/JAK2/TYK2 [59], and the phosphorylated STAT1 is transported into the nucleus with the form of homodimer and activate GAS (gamma interferon activation site) and ISRE (IRF9 and STAT2 dependent response). With the identical tendency of STAT1 mRNA level, OAS1 and IRF7 (identified by GEO2R and the limma R package) are important ISGs [60, 61]. Overall, STAT1, OAS1, and IRF7 in the IFN-JAK-STAT pathway are oppositely affected in IDC and SLE.

Immune responses work during and/or after tumorigenesis and autoimmune genesis anisotropically. Inadequate immunity caused by poor immune surveillance leads to tumorigenesis, while excessive immunity due to breakdown of immune tolerance causes autoimmune genesis. Indeed, multiple shared molecular mechanisms with opposite changes were reported to explain both tumorigenesis and autoimmune genesis such as TGF-β/Smad signaling and nuclear factor kappa-B (NF-κB) signaling. The TGF-β/Smad axis works both on cancers and autoimmune diseases. For one thing, transforming growth factor beta (TGF-β) is a multifunctional regulator in cancer immunity. TGF-β is able to delay tumorigenesis by causing cell cycle arrest [62] and by reshaping tumor microenvironment (TME) [63] at early-stage. Furthermore, TGF-β promotes tumor progression through PI3K/AKT/mTOR pathway activation [64], basic fibroblast growth factor (bFGF) signaling activation [65], the promotion of epithelial-mesenchymal transition (EMT) [66, 67], EMT-related immune surveillance [68, 69], and the inflammation induction by crosstalk with NF-κB signaling [70, 71] at late stage. The TGF-β/Smad signaling pathway works in a manner that, after the TGF-β binds with the corresponding proteins, the phosphorylated TGF-β receptors phosphorylate Smad2/3 for recruiting Smad4 to Smad2/3 complex for nuclear entry [64]. Hence, Smad3 promotes cancer by influencing reshaping of TME [63]. Smad3-silence strategy is an enhanced immunotherapy in cancers [72], and targeting Smad3/Smad7 enhances NK cells’ functions in anti-cancer immunity [73]. For another, the TGF-β/Smad signaling pathway is also considered as a crucial regulatory pathway in autoimmunity. The phosphorylated TGF-β receptors induce inhibitory Smad7 for a inhibition to the phosphorylation of Smad2/3 as a negative feedback [74]. Deficient Smad7 associates with TGF-β/Smad3-IL-6 signaling activation and Th17-induced immune responses [75], which lead to series of inflammatory damages. And it has been confirmed that targeting Smad7 strategy could ameliorate autoimmune renal disease in animal models [76]. NF-κB signaling is considered as an another example of pathways that both work in cancers and autoimmune diseases [77, 78]. For one thing, NF-κB signaling is being one of the pathways associated with immune system malfunctions [79, 80], which leads to tumorigenesis [81–87]. Study have shown that posttranslational modifications of NF-κB such as ubiquitination [88] and phosphorylation [84], are vital in regulating its activity, especially in necroptosis, autophagy, and apoptosis [83]. Such activity suggests an ubiquitous activity in cancer development, it is shown that not only the protein expression level but also the posttranslational modifications can be changed in different types of cancers. Phosphorylated NF-κB at Ser536 increases the pancreatic cancer cell motility [84]. Poly-ubiquitination of NF-κB signaling components such as IKB and IKKs can activate the nuclear transportation of NF-κB [80, 88]. In addition to the influence in protein levels, NF-κB signaling involves in tumorigenesis on genetic levels such as epigenetic effects (H3K9me3) and the stability of tumor suppressor (PTEN) [89, 90]. For another, NF-κB signaling contributes to autoimmune genesis and progression via the imbalance of multiple immune components. The canonical p50/p65 NF-κB signaling cascades are able to activate downstream immune responses of T cell receptors (TCRs), B cell receptors (BCRs), toll-like receptors(TLRs), and pro-inflammatory cytokines (TNFα and IL-1β) [91]. NF-κB signaling cascades contribute to the functional maturation of DCs in innate immune system, and they also could decelerate the activation of autoreactive T cells and favor the survival of B cells in innate adaptive system [92, 93]. Moreover, genetic associations cause autoimmune disorders by driving NF-κB signaling cascades [91].

We would like to highlight the strengths in choosing bioinformatic methods and disease models in this study. As for methods, we conducted differential analysis with multiple GEO databases (five IDC and five SLE) with GEO2R, the limma R package (after batch effect elimination), and WGCNA. With limitations of potential deviations from one of the methods, numerous studies tend to be conducted with one of the three bioinformatics tools. Although there are similarities between GEO2R and the limma R package, our study is able to render an available comparison among them, which is inspiring for data mining processes in the future. From our results, different genes were identified from the three modes. We speculated that the possible reason of different outcome of DEGs/hub genes among three bioinformatics methods was the batch effects. There should be batch effects in method of GEO2R, because the data were from different databases while the combined data were eliminated batch effects. However, those genes enriched almost at the similar biological events/pathways according to functional enrichment analysis (Fig. 2G, Fig. 3I, Fig. 3J,and Fig. 4I), which indicated the three methods are reliable and are able to do mutual authentications for one another. As for the disease model, breast cancer is the most common type of cancer among female population, and IDC is the most common type of breast cancer with high incidence in females [22, 24, 94, 95], while SLE is prevalent in a large population with a much higher incidence in females than males [26, 27]. Furthermore, both IDC and SLE are prevalent among females of childbearing age [22, 24, 26, 27, 94, 95]. Hence, since there are comparable physical conditions between the subjects with IDC and SLE, we believed that IDC and SLE are ideal and reasonable models to study the discrepancies and the commonalities between tumorigenesis and autoimmune genesis.

From this study, the mRNA levels of STAT1, OAS1, OASL, and PML were found to be differentially expressed in both IDC and SLE by using three different bioinformatics tools of GEO2R, the limma R package and WGCNA. From the combined databases in this study, the mRNA levels of STAT1 and OAS1 were increased in IDC while reduced in SLE manifesting the opposite expression tendency across cancer and autoimmune disease. According to pathway analysis, both IDC and SLE were correlated with the changes of multiple components involved in the IFN-JAK-STAT signaling pathway. We believe that the STAT1 and OAS1-associated IFN-JAK-STAT signaling pathway could explain the commonalities during tumorigenesis and autoimmune genesis. We hope that it could be beneficial for precion medicine in the future.

## Conclusion

In conclusion, we explored the resemblances of gene expression changes during tumorigenesis and autoimmune genesis and their effects on the immune response for the maintaining of immune homeostasis. The expression levels of STAT1 and OAS1 manifest the opposite expression tendency across cancer and autoimmune disease. They are components in the IFN-JAK-STAT signaling pathway related to both tumorigenesis and autoimmune genesis. STAT1 and OAS1-associated IFN-JAK-STAT signaling may explain the commonalities during tumorigenesis and autoimmune genesis and render significant information for more precise treatment from the point of immune homeostasis.

## Supplementary Information

**Additional file 1: Table S1.** Top 10 genes in network within 27 DEGs ranked by Betweenness method. **Table S2.** Top 10 genes in network within 58 DEGs ranked by Betweenness method. **Table S3.** Top 10 genes in network within 96 Hub genes ranked by Betweenness method. **Table S4.** The 541 IPA pathways identified from the 777 genes in IDC. **Table S5.** The 80 IPA pathways identified from the 24 genes in SLE.

**Additional file 2: Table S6.** Defined threshold of GEO2R

**Additional file 3: Table S7.** Defined threshold of the limma package

**Additional file 4: Figure S1.** The PPI network and functional enrichment analysis of crucial genes identified at least two modes. (A) PPI network of genes identified at least two modes (5 + 4 + 15). (B) GO enrichment analysis based on 24 genes shown in (A). (C) KEGG enrichment analysis based on 24 genes shown in (A).

**Additional file 5: Figure S2.** KEGG pathway analysis based on the 777 genes from IDC and the 24 genes from SLE. (A) KEGG pathway analysis based on the 777 genes from IDC (B) KEGG pathway analysis based on the 24 genes from SLE.

**Additional file 6: Figure S3.** Sample Tree showing the IDC patients.

**Additional file 7: Figure S4.** Sample Tree showing the SLE patients.

## Data Availability

The datasets generated during and/or analyzed during the current study are available in the GEO database repository, [https://www.ncbi.nlm.nih.gov/geo/].

## Abbreviations

IDC: Invasive ductal carcinoma
SLE: Systemic lupus erythematosus
GEO: Gene Expression Omnibus
PCA: Principal Component Analysis
DEGs: Differential expressed genes
FC: Fold change
PPI: Protein-protein interaction
BC: Betweenness centrality
GO: Gene Ontology
BC: Betweenness centrality
BP: Biological process
CC: Cellular component
MF: Molecular function
KEGG: Kyoto Encyclopedia of Genes and Genomes
IPA: Ingenuity pathway analysis
WGCNA: Weighted gene co-expression network analysis
kME: Module eigengene-based connectivity
MEs: Module eigengenes
STAT1: Signal transducer and activator of transcription 1
OAS1: 2'-5'-Oligoadenylate Synthetase 1
OASL: 2'-5'-Oligoadenylate Synthetase Like
PML: PML Nuclear Body Scaffold
STAT2: Signal transducer and activator of transcription 2
STAT3: Signal transducer and activator of transcription 3
IFNs: Interferons
IFNGR1: Interferon gamma receptor 1
IFNGR2: Interferon gamma receptor 2
IFNAR1: Interferon alpha and beta receptor subunit 1
IFNAR2: Interferon alpha and beta receptor subunit 2
IFNLR1: Interferon lambda receptor 1
IL-10RB: Interleukin 10 receptor subunit beta
TYK2: Tyrosine kinase 2
JAK1: Janus kinase 1
JAK2: Janus kinase 2
JAK3: Janus kinase 3
IRF7: Interferon regulatory factor 7
IFR9: Interferon regulatory factor 9
GAS: Gamma interferon activation site
ISRE: IFN-stimulated response element
SLIDAI: Systemic Lupus Erythematosus Disease Activity Index
ACC: Adrenocortical carcinoma
BLCA: Bladder urothelial carcinoma
BRCA: Breast invasive carcinoma
CESC: Cervical squamous cell carcinoma and endocervical adenocarcinoma
CHOL: Cholangio carcinoma
COAD: Colon adenocarcinoma
DLBC: Lymphoid neoplasm diffuse large B-cell lymphoma
ESCA: Esophageal carcinoma
GBM: Glioblastoma multiforme
HNSC: Head and neck squamous cell carcinoma
KICH: Kidney chromophobe
KIRC: Kidney renal clear cell carcinoma
KIRP: Kidney renal papillary cell carcinoma
LAML: Acute myeloid leukemia
LGG: Brain lower grade glioma
LIHC: Liver hepatocellular carcinoma
LUAD: Lung adenocarcinoma
LUSC: Lung squamous cell carcinoma
MESO: Mesothelioma
OV: Ovarian serous cystadenocarcinoma
PAAD: Pancreatic adenocarcinoma
PCPG: Pheochromocytoma and paraganglioma
PRAD: Prostate adenocarcinoma
READ: Rectum adenocarcinoma
SARC: Sarcoma
SKCM: Skin cutaneous melanoma
STAD: Stomach adenocarcinoma
TGCT: Testicular germ cell tumors
THCA: Thyroid carcinoma
THYM: Thymoma
UCEC: Uterine corpus endometrial carcinoma
UCS: Uterine carcinosarcoma
UVM: Uveal melanoma
SSc: Systemic sclerosis
RA: Rheumatoid arthritis
T1D: Type 1 diabete
SjS: Sjögren's syndrome
PTPRC: Protein tyrosine phosphatase receptor type C
CEACAM1: Carcinoembryonic antigen-related cell adhesion molecule 1
FCGR3B: Fc Gamma Receptor IIIb
ITGAX: Integrin subunit alpha X
IL3RA: Interleukin 3 receptor subunit alpha
NCAM1: Neural cell adhesion molecule 1
MS4A1: Membrane spanning 4-domains A1
