# Supplementary material for: Bioinformatics analyses of combined databases identify shared differentially expressed genes in cancer and autoimmune disease
Source: J Transl Med. 2023 Feb 10;21:109. doi: 10.1186/s12967-023-03943-9 (PMC9921081; doi:10.1186/s12967-023-03943-9)
Supplement: Supplementary file 2 — Additional file 2: Table S6. Defined threshold of GEO2R [file 12967_2023_3943_MOESM2_ESM.docx]

| Table S1. Defined threshold of GEO2R | | | |
| --- | --- | --- | --- |
| \|logFC\| | >0.5 | >1 | >2 |
| adj.P.Val | <0.05 | <0.05 | <0.05 |
| IDC | 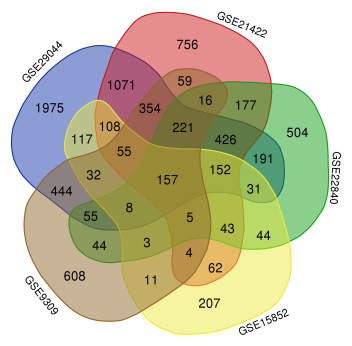 | 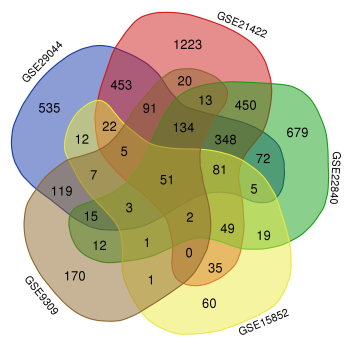 | 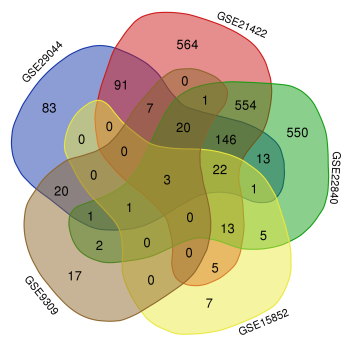 |
| SLE | 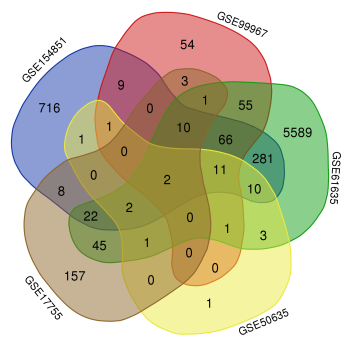 | 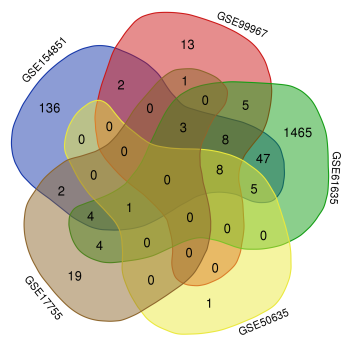 | 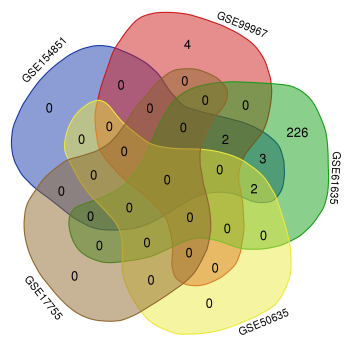 |

IDC, Invasive ductal carcinoma; SLE, Systemic lupus erythematosus; FC, Fold change
