# Supplementary material for: Bioinformatics analyses of combined databases identify shared differentially expressed genes in cancer and autoimmune disease
Source: J Transl Med. 2023 Feb 10;21:109. doi: 10.1186/s12967-023-03943-9 (PMC9921081; doi:10.1186/s12967-023-03943-9)
Supplement: Supplementary file 3 — Additional file 3: Table S7. Defined threshold of the limma package [file 12967_2023_3943_MOESM3_ESM.docx]

| Table S7. Defined threshold of the limma package | | | | | | | | | |
| --- | --- | --- | --- | --- | --- | --- | --- | --- | --- |
| Merged IDC | | | | | Merged SLE | | | | |
| \|logFC\| | adj.P.Val | DOWN | Not sig | UP | \|logFC\| | adj.P.Val | DOWN | Not sig | UP |
| >0.5 | <0.05 | 5681 | 256 | 5012 | >0.5 | <0.05 | 9 | 3788 | 60 |
| >1 | <0.05 | 5681 | 256 | 5012 | >1 | <0.05 | 0 | 3844 | 13 |
| >2 | <0.05 | 5681 | 257 | 5011 | >2 | <0.05 | 0 | 3857 | 0 |
| >3 | <0.05 | 5676 | 269 | 5004 | >3 | <0.05 | 0 | 3857 | 0 |
| >0.5 | <0.1 | 5708 | 204 | 5037 | >0.5 | <0.1 | 9 | 3788 | 60 |
| >1 | <0.1 | 5708 | 204 | 5037 | >1 | <0.1 | 0 | 3844 | 13 |
| >2 | <0.1 | 5706 | 208 | 5035 | >2 | <0.1 | 0 | 3857 | 0 |
| >3 | <0.1 | 5699 | 222 | 5028 | >3 | <0.1 | 0 | 3857 | 0 |

IDC, Invasive ductal carcinoma; SLE, Systemic lupus erythematosus; FC, Fold change
