# Supplementary figures and images for: Bioinformatics analyses of combined databases identify shared differentially expressed genes in cancer and autoimmune disease
Source: J Transl Med. 2023 Feb 10;21:109. doi: 10.1186/s12967-023-03943-9 (PMC9921081; doi:10.1186/s12967-023-03943-9)

### Cluster Dendrogram

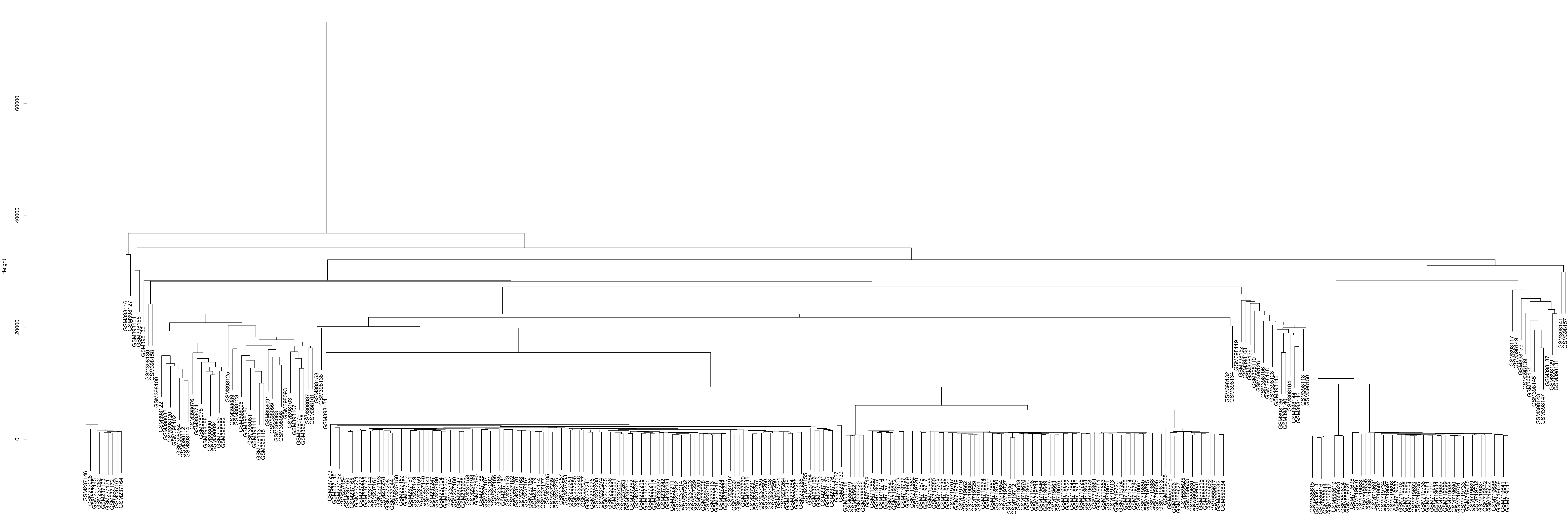

Supplement: Supplementary file 6 — Additional file 6: Figure S3. Sample Tree showing the IDC patients. [file 12967_2023_3943_MOESM6_ESM.pdf]
