# Supplementary material for: Bioinformatics analyses of combined databases identify shared differentially expressed genes in cancer and autoimmune disease
Source: J Transl Med. 2023 Feb 10;21:109. doi: 10.1186/s12967-023-03943-9 (PMC9921081; doi:10.1186/s12967-023-03943-9)
Supplement: Supplementary file 7 — Additional file 7: Figure S4. Sample Tree showing the SLE patients. [file 12967_2023_3943_MOESM7_ESM.pdf]

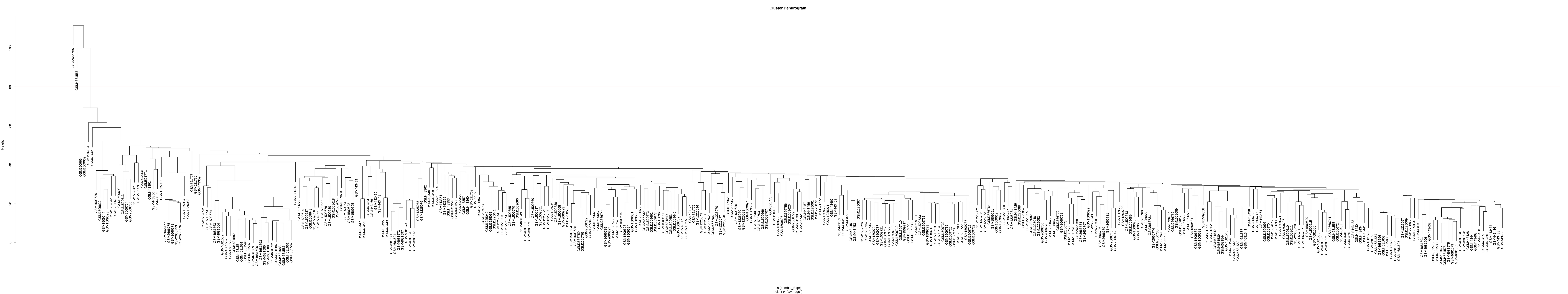
Cluster Dendrogram
Height
0
20
40
60
80
100
dist(combat_Expr)
hclust (*, "average")
